# Exploring the Relationship Between Insulin Resistance, Liver Health, and Restrictive Lung Diseases in Type 2 Diabetes

**DOI:** 10.3390/jpm15080340

**Published:** 2025-08-01

**Authors:** Mani Roshan, Christian Mudrack, Alba Sulaj, Ekaterina von Rauchhaupt, Thomas Fleming, Lukas Schimpfle, Lukas Seebauer, Viktoria Flegka, Valter D. Longo, Elisabeth Kliemank, Stephan Herzig, Anna Hohneck, Zoltan Kender, Julia Szendroedi, Stefan Kopf

**Affiliations:** 1 Department of Endocrinology, Diabetology, Metabolism and Clinical Chemistry (Internal Medicine 1), Heidelberg University Hospital, 69120 Heidelberg, Germany; mani.roshan@med.uni-heidelberg.de (M.R.); christian.mudrack@med.uni-heidelberg.de (C.M.); alba.sulaj@med.uni-heidelberg.de (A.S.); ekaterina.rauchhaupt@med.uni-heidelberg.de (E.v.R.); thomas.fleming@med.uni-heidelberg.de (T.F.); lukas.schimpfle@med.uni-heidelberg.de (L.S.); lukas.seebauer@med.uni-heidelberg.de (L.S.); viktoria.flegka@med.uni-heidelberg.de (V.F.); elisabeth.kliemank@med.uni-heidelberg.de (E.K.); annalena.hohneck@med.uni-heidelberg.de (A.H.); zoltan.kender@med.uni-heidelberg.de (Z.K.); julia.szendroedi@med.uni-heidelberg.de (J.S.); 2 German Centre for Diabetes Research (DZD), Helmholtz Centre Munich, 85764 Neuherberg, Germany; stephan.herzig@helmholtz-muenchen.de; 3 Longevity Institute, School of Gerontology, Department of Biological Sciences, University of Southern California, Los Angeles, CA 90089, USA; vlongo@usc.edu; 4 FIRC Institute of Molecular Oncology, Italian Foundation for Cancer Research Institute of Molecular Oncology, 20139 Milan, Italy; 5 Institute for Diabetes and Cancer, Helmholtz Center Munich, 85764 Neuherberg, Germany; 6 Joint Heidelberg-IDC Translational Diabetes Program, Internal Medicine I, Heidelberg University Hospital, 69120 Heidelberg, Germany

**Keywords:** restrictive lung disease, type 2 diabetes, insulin resistance, intermittent fasting diet, fasting-mimicking-diet, MASLD, FLI, HOMA-IR, Mediterranean diet

## Abstract

**Background**: Restrictive lung disease (RLD) is a potential complication in type 2 diabetes (T2D), but its relationship with insulin resistance and liver-related metabolic dysfunction remains unclear. This study evaluated the association between lung function and metabolic markers in T2D and retrospectively assessed whether metabolic improvements from dietary intervention were accompanied by changes in lung function. **Methods**: This cross-sectional analysis included 184 individuals (101 with T2D, 33 with prediabetes, and 50 glucose-tolerant individuals). Lung function parameters—vital capacity (VC), total lung capacity by plethysmography (TLC-B), and diffusion capacity for carbon monoxide (TL_CO_)—were assessed alongside metabolic markers including HOMA2-IR, fatty liver index (FLI), NAFLD score, and Fibrosis-4 index (FIB-4). In a subset of 54 T2D participants, lung function was reassessed after six months following either a fasting-mimicking diet (FMD, n = 14), Mediterranean diet (n = 13), or no dietary intervention (n = 27). **Results**: T2D participants had significantly lower VC and TLC-B compared to glucose-tolerant and prediabetic individuals, with 18–21% falling below clinical thresholds for RLD. Lung volumes were negatively correlated with HOMA2-IR, FLI, NAFLD score, and FIB-4 across the cohort and within the T2D group. Although the FMD intervention led to significant improvements in HOMA2-IR and FLI, no corresponding changes in lung function were observed over the six-month period. **Conclusions**: Restrictive lung impairment in T2D is associated with insulin resistance and markers of liver steatosis and fibrosis. While short-term dietary interventions can improve metabolic parameters, their effect on lung function may require a longer duration or additional interventions and targeted follow-up. These findings highlight the relevance of pulmonary assessment in individuals with metabolic dysfunction.

## 1. Introduction

Restrictive lung disease (RLD) is increasingly recognized as a potential complication in individuals with type 2 diabetes (T2D). Characterized by reduced lung volumes and impaired respiratory function, RLD contributes to poorer health outcomes in T2D; however, its risk factors remain inadequately explored [1]. Previous research suggests a link between RLD and metabolic disorders, but the specific contributions of insulin resistance and liver health indicators to RLD development remain unclear [2]. Notably, long-term T2D and albuminuria have previously been described as independent risk factors for RLD [3].

Insulin resistance, a hallmark of T2D, is associated with various systemic complications, including metabolic dysfunction-associated steatotic liver disease (MASLD). MASLD is prevalent in T2D and has been linked to chronic inflammation and oxidative stress [4,5,6,7,8], both of which are implicated in respiratory impairments, such as RLD [9,10,11]. Despite these associations, limited studies have investigated whether insulin resistance and liver health directly correlate with the onset or severity of RLD in individuals with T2D.

Additional contributors to reduced lung function include cigarette smoking, a well-established source of reactive oxygen species that exacerbate pulmonary damage [12,13,14,15,16,17,18,19]. In individuals with T2D, poor glycemic control has also been linked to impaired lung function, with elevated HbA1c levels associated with reduced RLD parameters [20,21,22,23]. Together, these findings suggest that both lifestyle-related exposures and metabolic dysregulation may contribute to the pathogenesis of RLD in T2D.

Lifestyle interventions, particularly dietary modifications, are known to improve insulin sensitivity and reduce markers of liver health [24,25,26,27,28,29]. Diets that mimic fasting, such as a fasting-mimicking diet (FMD), have shown promise in reducing insulin resistance and MASLD markers in T2D populations [30,31,32].

In this study, we conducted a retrospective analysis of data from prior studies where lung function was assessed. The primary objective was to evaluate the association between RLD and T2D, with a particular focus on the roles of insulin resistance and liver health markers. Additionally, we aimed to determine whether dietary interventions, specifically an FMD, could improve RLD-related outcomes by targeting metabolic dysfunction.

## 2. Materials and Methods

### 2.1. Study Design and Participants

This retrospective analysis used data from two sources to address distinct research questions. First, data from the Heidelberg Study on Diabetes and Complications (Heist-DiC) and a previously published periodic FMD cohort [31,33,34] were analyzed to investigate the association between RLD and T2D. The Heist-DiC cohort included 184 participants: 101 with T2D, 33 with prediabetes (PRED), and 50 normal glucose-tolerant (NGT) individuals. Glucose tolerance status was defined based on standard clinical criteria, including fasting glucose, 120 min oral glucose tolerance test (oGTT), HbA1c, and confirmed diabetes diagnosis. Baseline assessments of lung function and metabolic parameters were evaluated for all participants to explore potential links between RLD and T2D (Supplementary Figure S1).

To assess whether dietary interventions could improve RLD, follow-up data from the FMD intervention study was used [31]. In this study, participants in the FMD group (n = 14) followed a structured five-day-per-month ketogenic diet simulating fasting, while the Mediterranean diet group (n = 13) received dietary counseling. The no-intervention group consisted of 27 matched T2D participants from the Heist-DiC cohort, selected based on age, body mass index (BMI), smoking status, and sex. Baseline and six-month follow-up assessments in the FMD group and a 12-month follow-up assessment in the Heist-DiC cohort included both lung and metabolic parameters from all participants (Supplementary Figure S1).

These studies received ethical approval from the Heidelberg University ethics committee (approval codes S-383/2016 and S-682/2016). Written informed consent was obtained from all participants, and the study adhered to the Declaration of Helsinki.

### 2.2. Lung Function Testing and Metabolic Parameters

Primary measurements for assessing RLD included vital capacity (VC), total lung capacity (TLC-B), and diffusion capacity (TL_CO_). Lung function testing was performed in accordance with American Thoracic Society guidelines [35,36] using the PowerCube Body+ device (Ganshorn Medizin Electronic GmbH, Niederlauer, Germany). Oxygen saturation (SpO_2_) was measured using the Pulox^®^ PO-250 device (Novodion GmbH, Cologne, Germany). Predicted values for VC, TLC-B, and TL_CO_ were calculated using standard reference equations embedded in the Ganshorn system, adjusted for age, sex, and body size. For TL_CO_, values for all participants were additionally corrected for current hemoglobin levels. Lung function was considered reduced when VC, TLC-B, or TL_CO_ was <80% predicted, consistent with a restrictive ventilatory pattern [3,36,37,38]. As FEV1/VC was not assessed, restrictive physiology was inferred from lung volumes alone.

Blood samples were collected from all participants after overnight fasting and processed immediately in the Central Laboratory of the University Hospital of Heidelberg under standardized conditions. Whole-body insulin sensitivity was determined using HOMA2-IR, calculated from fasting glucose and C-peptide levels using the Oxford HOMA calculator [39]. Liver steatosis and fibrosis were evaluated using non-invasive scoring systems based on clinical characteristics and laboratory parameters, including the NAFLD score (BMI, diabetes status, aspartate aminotransferase (AST), alanine aminotransferase (ALT), and platelet count), the fatty liver index (FLI) (BMI, waist circumference, triglycerides, and gamma-glutamyl transferase (GGT)), and the fibrosis-4 (FIB-4) index (age, AST, ALT, and platelet count) [40]. Smoking status, medication use, and diabetes complications were recorded.

### 2.3. Statistical Methods

Descriptive statistics were calculated for baseline characteristics. Group comparisons were performed using one-way ANOVA or Kruskal–Wallis tests for continuous variables and Fisher’s tests for categorical data, as appropriate. Associations between lung function parameters and metabolic markers were assessed using Spearman’s rank correlation and multiple linear regression. Correlation analyses were conducted both across the full cohort and within the T2D subgroup to assess within-group effects. A significance level of α = 0.05 was applied for all tests. Statistical analysis was conducted in Prism 7–10 (GraphPad Software Inc., La Jolla, CA, USA).

## 3. Results

### 3.1. Baseline Characteristics

Baseline characteristics for the three participant groups (NGT, PRED, and T2D) are summarized in Table 1. Participants with T2D were older, more likely to be male, and had a higher BMI compared to those with NGT or PRED. They also exhibited significantly higher markers of insulin resistance (HOMA2-IR) and impaired glycemic control (HbA1c). Liver dysfunction markers, including the NAFLD and FIB-4 scores and FLI, were markedly elevated, reflecting an increased risk of fibrosis and steatosis. Statin use and antidiabetic medication (oral or insulin) were more common in the T2D group, consistent with a higher burden of metabolic disease.

Pulmonary comorbidities were infrequent across all groups. Asthma and COPD were reported in 6.5% of participants, while obstructive sleep apnea syndrome (OSAS) was more prevalent in the T2D group (10.9%) and was commonly managed with continuous positive airway pressure (CPAP) therapy (9.9%). These conditions may contribute to individual variability in lung function but were not sufficient to account for group-level differences. Smoking rates were low and comparable across the three groups (6.0% in NGT, 6.1% in PRED, and 5.9% in T2D), suggesting that observed differences in lung function were unlikely to be driven by tobacco exposure.

**Figure 1:**
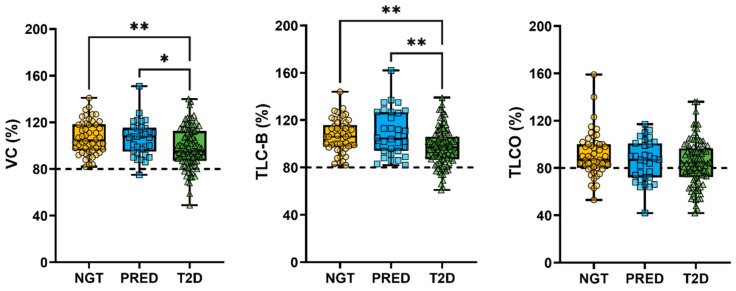
Boxplots with overlaid individual data points showing lung function parameters—VC, TLC-B, and TL_CO_—in NGT (yellow circles), PRED (blue squares), and T2D (green triangles) groups. Dashed horizontal lines indicate the clinical thresholds for restrictive lung disease at < 80%. Statistically significant group differences are indicated as follows: * *p* < 0.05; ** *p* < 0.01.

A higher prevalence of lung disease was observed in participants with T2D, despite similar smoking rates across the groups. Both VC and TLC-B were significantly lower in individuals with T2D compared to those with PRED and NGT, although values remained within the reference range. TL_CO_ did not differ significantly between groups (Figure 1).

A greater proportion of T2D participants fell below the diagnostic thresholds for RLD: VC < 80% (12/89; 12%) and TLC-B < 80% (14/87; 14%)—compared to NGT (VC: 0/50; 0%; TLC-B: 0/50; 0%) and PRED (VC: 1/32; 3%; TLC-B: 1/32; 3%). These group differences were statistically significant (Fisher’s exact test: VC = 0.0086; TLC-B = 0.0039). Reductions in TL_CO_ (<80%) were also observed in T2D (43/58; 43%) and PRED (13/20; 39%), but were not specific to either group, as 13/37 (26%) of NGT participants also fell below this threshold (Fisher’s exact test: TL_CO_ = 0.1289). These findings support an association between metabolic status and restrictive lung physiology, with impaired gas transfer potentially emerging earlier in the disease continuum.

### 3.2. Association Between Metabolic Markers and Lung Function

**Figure 2:**
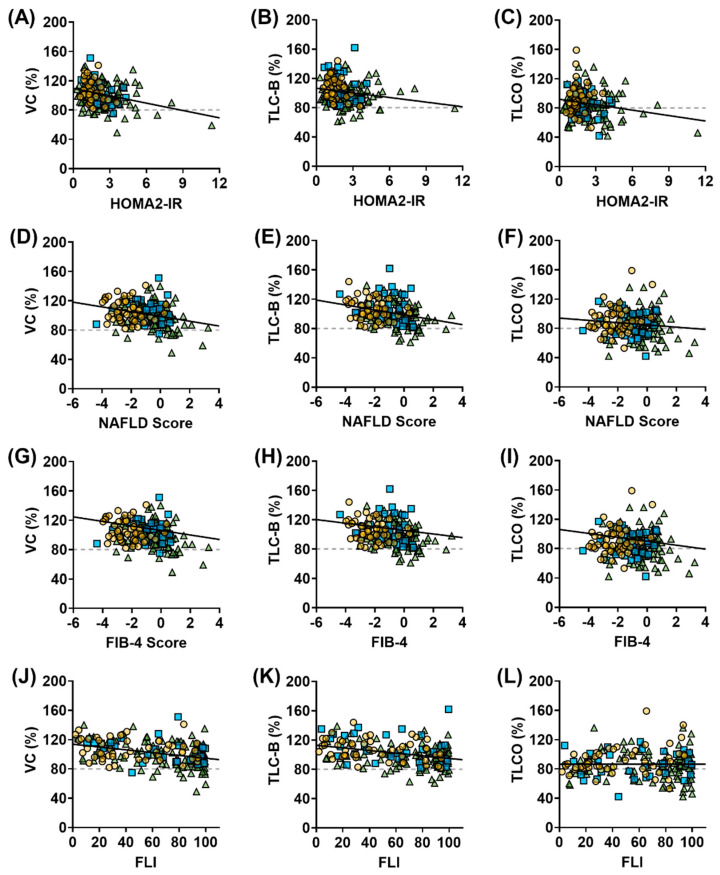
Scatter plots showing the relationships between metabolic markers—HOMA2-IR (**A**–**C**), NAFLD score (**D**–**F**), FIB-4 (**G**–**I**), and FLI (**J**–**L**)—and lung function parameters—VC, TLC-B, and TL_CO_. Each point represents an individual from the study cohort (n = 184): yellow circles indicate NGT participants, blue squares indicate PRED, and green triangles indicate individuals with T2D. Solid black lines represent linear regression trends; dashed gray lines indicate diagnostic thresholds for restrictive lung disease at <80%.

Spearman correlation analysis across the full cohort revealed significant negative associations between metabolic dysfunction and lung function parameters (Figure 2). HOMA2-IR was negatively correlated with VC (r = −0.242, *p* = 0.0009) and TLC-B (r = −0.212, *p* = 0.004), while its association with TL_CO_ was not statistically significant (r = −0.113, *p* = 0.128) (Figure 2A–C). The NAFLD score also correlated negatively with VC (r = −0.262, *p* = 0.0003) and TLC-B (r = −0.295, *p* < 0.0001), but not with TL_CO_ (r = −0.101, *p* = 0.173) (Figure 2D–F). FIB-4, as a fibrosis index without diabetes status as an impacting parameter, showed similar results with a weak correlation with VC (r = −0.138, *p* = 0.0627) and a weak negative correlation with TLC-B (r = −0.197, *p* = 0.0076) but not TL_CO_ (r = −0.072, *p* = 0.3361) (Figure 2G–I). FLI showed moderate negative correlations with VC (r = −0.370, *p* < 0.0001) and TLC-B (r = −0.323, *p* < 0.0001), but not with TL_CO_ (r = 0.009, *p* = 0.905) (Figure 2J–L).

To assess whether the relationship between metabolic dysfunction and lung function was more pronounced in T2D, a subgroup analysis was performed (Supplementary Table S1). In T2D, VC and TLC-B showed significant inverse associations with liver-related markers, including FLI, NAFLD score, FIB-4, liver stiffness, and Controlled Attenuation Parameter (CAP), an ultrasound-based method to quantify liver fat. For example, VC correlated negatively with FLI (r = −0.332), NAFLD score (r = −0.242), and liver stiffness (r = −0.318), while TLC-B was negatively correlated with NAFLD score (r = −0.211) and FIB-4 (r = −0.204). VC also correlated with FLI (r = −0.442) and CAP (r = −0.448) in PRED, while TLC-B correlated with FLI (r = −0.286) in NGT. TLCO was only correlated with FLI (r = 0.285) and CAP (r = 0.315) in NGT. Across the cohort, all lung parameters were negatively correlated with age and HbA1c; VC and TLC-B were also negatively associated with BMI. While VC and TLC-B consistently showed associations with hepatic indices and adiposity, TLCO showed no clear associations with metabolic parameters.

Given the consistency of association patterns across subgroups, and the collinearity of many metabolic predictors, multiple linear regression models were applied to the full cohort to assess the independent contributions of liver- and glucose-related parameters to lung function, while adjusting for age and BMI. These models included z-normalized age, BMI, HbA1c, HOMA2-IR, and FLI in combination with either NAFLD score or FIB-4.

FLI was the only significant predictor for VC (β = −4.661, *p* = 0.0195) and TLC-B (β = −6.254, *p* = 0.0018) in models using the NAFLD score, while TLCO was predicted by HOMA2-IR (β = −4.469, *p* = 0.0071). The results were comparable in models including FIB-4 (FLI for VC: β = −4.526, *p* = 0.0243; FLI for TLC-B: β = −6.310, *p* = 0.0018; HOMA2-IR for TLCO: β = −4.426, *p* = 0.0077). HbA1c was a significant predictor for TLC-B in the FIB-4 model (β = −3.029, *p* = 0.0192) and showed a trend in the NAFLD score model (β = −2.572, *p* = 0.0550). VC showed a trend toward association with HbA1c in the FIB-4 model (β = −2.376, *p* = 0.0581), while TLCO was not significantly impacted by HbA1c.

No significant correlations were found between HOMA2-IR and any lung parameter when restricting the analysis to T2D participants. These models did not include sex as a covariate due to the unequal sex distribution across groups, particularly in the T2D group, which limits interpretation of potential sex-specific effects. Together, these findings suggest that restrictive lung changes in T2D are linked more closely to hepatic dysfunction and systemic metabolic stress than to insulin resistance per se. TLCO appears to remain relatively preserved, supporting a distinction between restrictive physiology and microvascular/parenchymal damage.

### 3.3. Effects of Dietary Intervention on RLD

**Figure 3:**
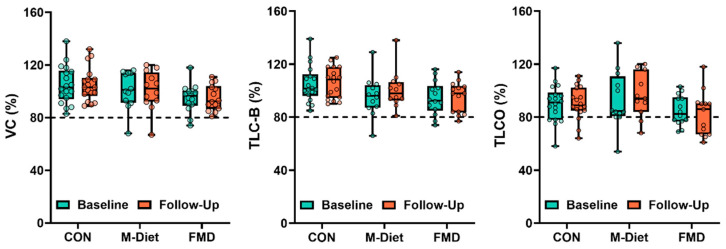
Lung function parameters before and after dietary intervention. Boxplots with overlaid individual values show VC, TLC-B, and TL_CO_ in participants assigned to FMD or M-Diet at baseline (teal) and after 6 months (orange). Data are shown as percent of predicted values. No significant changes in lung function were observed at group level for either intervention. Clinical thresholds for restrictive lung disease are indicated by dashed lines at <80%.

An FMD has previously been shown to significantly improve metabolic markers, such as HOMA-IR [24,31]. Retrospective analysis of this study revealed that neither the FMD nor Mediterranean diet led to significant changes in lung function parameters, including VC, TLC-B, or TL_CO_, during the six-month intervention period (Figure 3).

To explore whether individuals with pre-existing restrictive lung function responded differently, a subgroup analysis was conducted in participants with baseline values below diagnostic thresholds for VC, TLC-B, or TL_CO_ (<80%). In the FMD group, 2 of 14 participants had low VC and 2 had low TLC-B at baseline; all 4 showed normalization at follow-up. In the Mediterranean diet group, only one participant had reduced VC or TLC-B at baseline, limiting interpretation. No participants in the control group had reduced VC or TLC-B.

For TL_CO_, 6 of 13 participants in the FMD group had values below the threshold, with 1 normalizing by follow-up (17%). In the control group, 5 of 18 participants had low TL_CO_, of whom 3 improved (60%). In the Mediterranean diet group, 1 of 12 participants had TL_CO_ < 80%, with no improvement.

## 4. Discussion

This study aimed to evaluate the association between RLD and T2D, with a particular focus on the roles of insulin resistance and liver health. We observed a higher prevalence of reduced VC and TLC-B in individuals with T2D compared to those with NGT or PRED. Across the full cohort, metabolic markers including HOMA2-IR, FLI, NAFLD score, and FIB-4 were inversely correlated with lung volumes. These findings suggest that insulin resistance and hepatic dysfunction may contribute to impaired lung function in T2D. Although short-term dietary interventions significantly improved metabolic parameters, we did not observe corresponding improvements in lung function over the six-month period.

Our cross-sectional analysis demonstrated that participants with T2D exhibited reduced lung function parameters (VC, TLC-B, and TL_CO_) compared to both NGT individuals and those with PRED. In lung function measurements, the results were adjusted for age, sex, and BMI. Thus, minor weight differences between groups are not determinative of the reduced lung capacity observed in participants with T2D. Specifically, 12% of T2D participants had VC < 80% predicted and 14% had TLC-B < 80%, in contrast to 0% in the NGT and 3% in the PRED group, respectively. TL_CO_ < 80% was observed in 43% of T2D participants, but also in 39% of PRED and 26% of NGT individuals, suggesting that impaired gas transfer may arise earlier in the metabolic disease spectrum. These findings are consistent with previous studies showing an increased prevalence of pulmonary impairments in individuals with T2D [1,2,3,9]. Potential mechanisms underlying this association include systemic inflammation, oxidative stress, and impaired gas exchange, all of which are closely linked to insulin resistance and metabolic syndrome and have been implicated in pulmonary function decline [11].

The observed negative correlations between lung function parameters and markers of insulin resistance (HOMA2-IR) and liver fibrosis/steatosis (NAFLD score, FIB-4, and FLI) suggest that metabolic dysfunction may play a role in reducing lung function in T2D. Importantly, these associations were not solely driven by between-group differences across the glucose tolerance spectrum but were to some degree also evident within the T2D subgroup itself. This supports the hypothesis that systemic metabolic disturbances extend beyond traditional cardiovascular risk and are also associated with pulmonary complications in T2D. Mechanistically, fibrotic remodeling in T2D appears to involve shared pathways across metabolically active tissues, including the liver, lung, adipose tissue, and myocardium [41,42,43]. Chronic inflammation, dysregulated adipokine signaling, and advanced glycation end-products (AGEs) contribute to extracellular matrix deposition and loss of tissue compliance [41,42,43,44]. In the lung and kidney, emerging data suggest that metabolic stress may impair DNA repair capacity, leading to persistent DNA damage, cellular senescence, and profibrotic signaling [45]. Together, these mechanisms may underlie the observed reductions in lung volume and position RLD within the broader context of diabetes-related organ fibrosis.

The dietary intervention analysis revealed significant improvements in HOMA2-IR and FLI, and a reduction in senescence-associated secretory phenotype (SASP) mediators—a key mechanism in cellular senescence—in participants following an FMD. These findings are consistent with previous evidence on the metabolic benefits of fasting-mimicking and Mediterranean diets in T2D populations [31]. However, no significant changes were observed in lung function parameters (VC, TLC-B, and TL_CO_) over the six-month intervention period. Given the small sample sizes, these findings should be interpreted with caution. Nevertheless, the normalization of VC and TLC-B in a subset of FMD participants may reflect individual responsiveness and aligns with observed associations between lung volume and FLI in the regression analysis. In contrast, TL_CO_ appeared largely unaffected, in keeping with prior studies suggesting that gas transfer is less sensitive to metabolic or hepatic changes. Importantly, no deterioration in lung function was observed in any group, suggesting that short-term dietary interventions are at least not harmful in this context.

The lack of improvement in lung function despite metabolic gains suggests that a longer intervention period may be required to elicit structural or functional pulmonary changes. Alternatively, lung dysfunction in T2D may be driven by additional mechanisms that are not readily reversed through dietary modulation alone. These could include irreversible fibrotic remodeling, impaired tissue elasticity, or underlying microvascular damage. It is also possible that dietary interventions affect systemic inflammation and metabolic stress [46] before these effects translate into measurable pulmonary improvements. Future studies should evaluate the time course of lung function recovery in response to metabolic interventions and explore combination strategies—such as exercise training, anti-fibrotic agents, or senolytic therapies—to more effectively target lung dysfunction in T2D.

This study has several limitations. Most importantly, the cross-sectional design of the Heist-DiC cohort precludes any causal inference between metabolic markers and lung function. While we observed significant associations, these should be interpreted strictly as correlational. Moreover, potentially confounding factors were not fully controlled, and some relevant clinical information was unavailable. For instance, although we reported smoking status and comorbidity prevalence, detailed data on smoking exposure (e.g., pack-years), medication use (e.g., insulin, statins, and antihypertensives), physical activity, and coexisting pulmonary or cardiovascular conditions were not included in the correlation analyses. Additionally, although our regression models adjusted for age and BMI, they did not include sex, which is a limitation given the male predominance in the T2D group. This sex imbalance restricts the ability to examine sex-specific associations or generalize findings to women. Prior research has shown that sex influences lung function decline across the lifespan independent of disease status [47], and recent evidence indicates that females with restrictive spirometry patterns may experience greater symptom burden and impaired quality of life compared to males [48]. Future studies should aim for sex-balanced recruitment and conduct sex-stratified analyses to better characterize the metabolic–pulmonary axis in both men and women.

Second, although the FMD intervention successfully targeted metabolic improvements, lung function was not a predefined primary outcome. Consequently, the relatively short duration of the intervention may have been insufficient to detect meaningful changes in pulmonary parameters. Additionally, the small sample sizes in the dietary intervention groups limited the statistical power of subgroup analyses and constrained interpretation. To address these shortcomings, a prospective FMD study specifically targeting individuals with restrictive lung disease and incorporating a longer follow-up period would be necessary. Lastly, as participants were recruited from a single center in Germany and were predominantly middle-aged adults of European descent [49], the generalizability of these findings is limited and should be confirmed in more diverse populations across different ethnic and geographic backgrounds.

In conclusion, our findings indicate that T2D is associated with reduced lung function, and this association may be mediated, in part, by insulin resistance and liver fibrosis/steatosis. While dietary interventions can improve metabolic markers, their impact on lung function may require longer intervention periods. Clinicians should consider the potential impact of metabolic health on lung function in individuals with T2D and explore targeted interventions that address systemic metabolic dysfunction. Routine assessment of pulmonary function may be particularly relevant in individuals with longstanding T2D or coexisting hepatic steatosis, as such individuals may be at increased risk of subclinical lung restriction.

## Figures and Tables

**Table 1:** Baseline characteristics of participants in the Heist-DiC cohort.

Parameter	NGT	PRED	T2D	Sig.
No. of participants	50	33	101	
Age (years)	51.2 ± 7.4	59.9 ± 11.8	66.8 ± 8.0 ****/°°°	####
Sex (male/female)	21/29	18/15	68/33	#
BMI (kg/m^2^)	26.6 (24.1–30.8)	27.8 (24.7–33.6)	29.5 (26.0–32.9) *	#
Smoking (%)	3 (6.0%)	2 (6.1%)	6 (5.9%)	
Duration of diabetes (years)	–	–	13.0 (8.0–20.0)	
**Metabolic Markers**				
HbA1c (%)	5.4 (4.2–5.7)	5.7 (5.3–6.0)	7.0 (6.5–8.1) ****/°°°°	####
HOMA2-IR	1.3 (1.1–1.7)	1.8 (1.4–2.7) **	2.4 (1.7–3.6) ****	####
eGFR (mL/min/1.73 m^2^)	93.9 (88.1–99.4)	90.2 (84.0–106.1)	89.7 (74.2–97.8)	
uAlb/Cre (mg/g)	5.6 (3.4–10.3)	5.6 (3.3–11.3)	17.7 (7.2–45.0) ****/°°°°	####
**Liver Markers**				
NAFLD Score	–2.2 ± 1.0	–1.0 ± 1.2 ***	–0.4 ± 1.2 ****/°	####
Fibrosis-4 index (FIB-4)	1.1 (0.8–1.4)	1.0 (0.7–1.5)	1.3 (1.0–1.7) *	##
Fatty liver index (FLI)	38.5 (20.8–71.0)	62.3 (28.8–91.0)	79.6 (52.9–93.3) ****	####
**Lung Function**				
VC (% predicted)	104.5 (95.8–118.3)	108.0 (95.0–115.5)	95.0 (87.0–112.5) **	##
TLC-B (% predicted)	106.0 (97.8–115.8)	104.5 (94.0–126.8)	97.0 (87.0–106.0) **/°	###
TL_CO_ (% predicted)	87.0 (80.0–100.0)	87.0 (72.0–100.8)	84.0 (72.3–96.8)	
RLD (%)	13 (26.0%)	12 (38.7%)	48 (48.0%)	#
**Pulmonary Conditions**				
COPD (%)	1 (2.0%)	1 (3.0%)	3 (3.0%)	
Asthma (%)	9 (18.0%)	6 (18.2%)	9 (8.9%)	
OSAS (%)	3 (6.0%)	1 (3.0%)	11 (10.9%)	
CPAP therapy (%)	2 (4.1%)	0 (0.0%)	10 (9.9%)	
**Diabetes-Related Complications**				
Cardiovascular disease (%)	12 (24.5%)	14 (43.8%)	86 (85.2%)	####
Nephropathy (%)	3 (6.1%)	3 (9.1%)	39 (38.6%)	####
Neuropathy (%)	2 (4.0%)	6 (18.8%)	50 (51.6%)	####
**Medication Use**				
Statin (%)	6 (12.0%)	4 (12.1%)	64 (63.4%)	####
Oral antidiabetics (%)	–	–	78 (77.2%)	
Insulin (%)	–	–	28 (27.7%)	
Diet only/no meds (%)	–	–	18 (17.8%)	
Beta-blocker (%)	5 (10.2%)	5 (16.1%)	41 (41.0%)	####
Anticholinergics (%)	2 (4.1%)	1 (3.1%)	4 (4.1%)	
Beta2-antagonists (%)	4 (8.2%)	3 (9.4%)	9 (9.2%)	
Corticoids (%)	4 (8.2%)	2 (6.3%)	6 (6.1%)	

Baseline characteristics of participants in the Heist-DiC cohort, stratified for NGT, PRED, and T2D. Values are presented as median (25th–75th) percentile or mean ± standard deviation. * indicates a significance to NGT; **°** indicates a significance to PRED; # indicates a significant difference (Sig.) over all groups according to ANOVA or Fisher’s test.

## Data Availability

The original contributions presented in this study are included in the article/supplementary material. Further inquiries can be directed to the corresponding author(s).

## Supplementary Materials

The following supporting information can be downloaded at https://www.mdpi.com/article/10.3390/jpm15080340/s1. Figure S1. Participant selection flow chart for the Heist-DiC cohort and dietary intervention phase. Panel (A) shows the selection process for participants included in the study, including exclusion criteria and final group assignments (NGT, PRED, T2D). Panel (B) illustrates the breakdown of participants in the dietary intervention phase, detailing the allocation to different dietary groups (fasting-mimicking diet, Mediterranean diet, or no intervention). Table S1. Spearman correlation coefficients between lung function parameters (VC, TLC-B, TLCO) and clinical/metabolic variables in participants with NGT, PRED, T2D, and the full cohort. Variables include liver-related indices (FLI, NAFLD score, CAP, liver stiffness, FIB-4), anthropometric and glycemic markers (BMI, HbA1c, HOMA2-IR), and age. Statistically significant associations (*p* < 0.05) are shown in bold.
