# Supplementary material for: Exploring the Relationship Between Insulin Resistance, Liver Health, and Restrictive Lung Diseases in Type 2 Diabetes
Source: J Pers Med. 2025 Aug 1;15(8):340. doi: 10.3390/jpm15080340 (PMC12387788; doi:10.3390/jpm15080340)

Supplementary Figure 1. Participant selection flow chart for the Heist-DiC cohort and dietary intervention phase. Panel (**A**) shows the selection process for participants included in the study, including exclusion criteria and final group assignments (NGT, PRED, T2D). Panel (**B**) illustrates the breakdown of participants in the dietary intervention phase, detailing the allocation to different dietary groups (fasting-mimicking diet, Mediterranean diet, or no intervention).

(A)

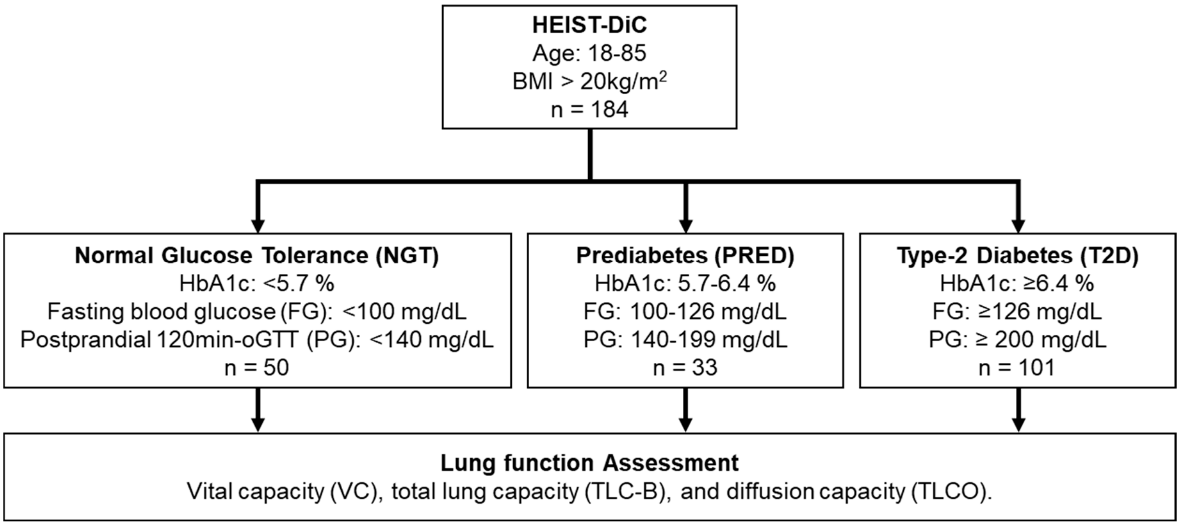

(B)

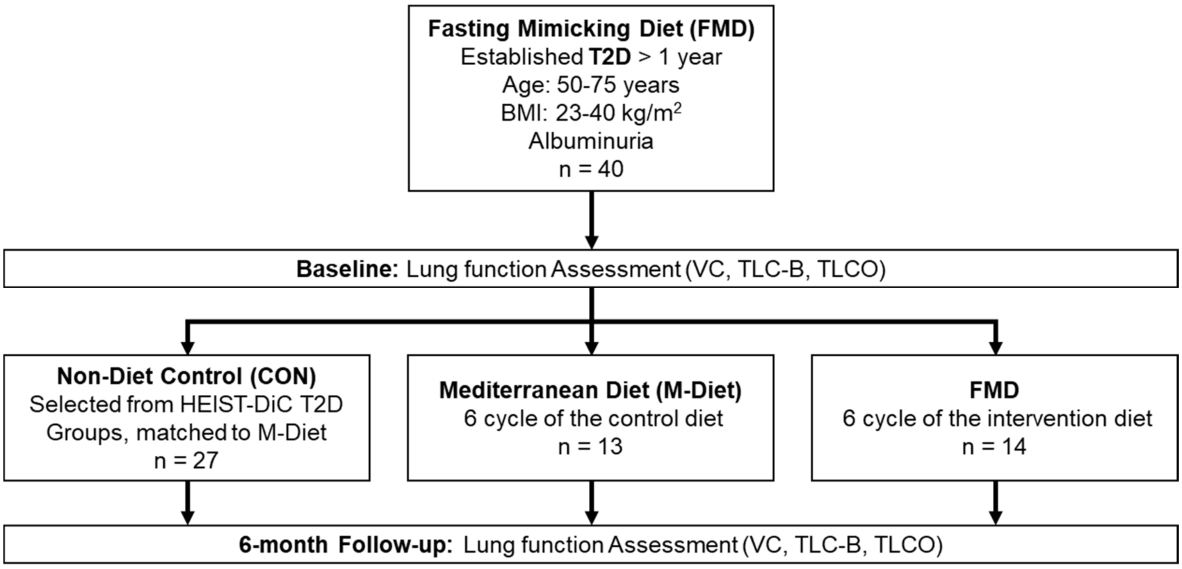

Supplement: Supplementary file 1 [file jpm-15-00340-s001.zip › jpm-3706548_Supplementary Figure 1.pdf]
