# Supplementary material for: Exploring the Relationship Between Insulin Resistance, Liver Health, and Restrictive Lung Diseases in Type 2 Diabetes
Source: J Pers Med. 2025 Aug 1;15(8):340. doi: 10.3390/jpm15080340 (PMC12387788; doi:10.3390/jpm15080340)
Supplement: Supplementary file 1 [file jpm-15-00340-s001.zip › jpm-3706548_Supplementary Table 1_Revised.pdf]

Supplementary Table 1. Spearman correlation coefficients between lung function parameters (VC, TLC-B, TLCO) and clinical/metabolic variables in participants with NGT, PRED, T2D, and the full cohort. Variables include liver-related indices (FLI, NAFLD score, CAP, liver stiffness, FIB-4), anthropometric and glycemic markers (BMI, HbA1c, HOMA2-IR), and age. Statistically significant associations ($p < 0.05$) are shown in bold.

Lung Parameter	Group	FLI	CAP	NAFLD Score	FIB-4	LS	BMI	Age	HbA1c	HOMA2-IR
**VC**	NGT	–0.193 (p=0.179)	–0.210 (p=0.144)	–0.245 (p=0.087)	–0.148 (p=0.306)	–0.253 (p=0.076)	–0.113 (p=0.434)	0.245 (p=0.086)	0.028 (p=0.848)	–0.309 (p=0.029)
	PRED	**–0.442 (p=0.010)**	**–0.448 (p=0.015)**	–0.092 (p=0.611)	0.089 (p=0.623)	–0.227 (p=0.227)	**–0.482 (p=0.005)**	–0.051 (p=0.780)	–0.023 (p=0.898)	**–0.521 (p=0.002)**
	T2D	**–0.332 (p=0.001)**	–0.162 (p=0.118)	**–0.242 (p=0.015)**	–0.141 (p=0.159)	**–0.318 (p=0.002)**	–0.182 (p=0.069)	–0.177 (p=0.077)	–0.208 (p=0.037)	–0.076 (p=0.450)
	All	**–0.370 (p<0.0001)**	**–0.296 (p<0.0001)**	**–0.262 (p=0.0003)**	–0.138 (p=0.063)	**–0.324 (p<0.0001)**	**–0.242 (p=0.0009)**	–0.190 (p=0.0097)	**–0.291 (p<0.0001)**	**–0.242 (p=0.0009)**
**TLC-B**	NGT	**–0.286 (p=0.044)**	–0.265 (p=0.064)	–0.201 (p=0.162)	–0.143 (p=0.322)	–0.160 (p=0.266)	–0.183 (p=0.203)	0.240 (p=0.093)	0.077 (p=0.593)	–0.205 (p=0.154)
	PRED	–0.305 (p=0.090)	–0.263 (p=0.176)	–0.240 (p=0.186)	0.021 (p=0.910)	–0.193 (p=0.316)	**–0.375 (p=0.034)**	–0.074 (p=0.688)	–0.141 (p=0.441)	–0.270 (p=0.135)
	T2D	–0.163 (p=0.106)	–0.108 (p=0.305)	**–0.211 (p=0.036)**	**–0.204 (p=0.042)**	–0.189 (p=0.067)	–0.040 (p=0.694)	**–0.310 (p=0.002)**	–0.183 (p=0.068)	–0.042 (p=0.681)
	All	**–0.323 (p<0.0001)**	**–0.268 (p=0.0004)**	**–0.295 (p<0.0001)**	**–0.197 (p=0.0076)**	**–0.242 (p=0.0013)**	**–0.186 (p=0.012)**	**–0.265 (p=0.0003)**	**–0.320 (p<0.0001)**	**–0.212 (p=0.004)**
**TLCO**	NGT	**0.285 (p=0.045)**	**0.315 (p=0.026)**	0.139 (p=0.336)	0.107 (p=0.460)	0.018 (p=0.900)	0.210 (p=0.143)	0.055 (p=0.706)	–0.022 (p=0.880)	0.227 (p=0.113)
	PRED	0.016 (p=0.929)	–0.223 (p=0.254)	0.016 (p=0.929)	0.127 (p=0.490)	0.259 (p=0.175)	–0.054 (p=0.769)	–0.139 (p=0.447)	0.133 (p=0.467)	–0.331 (p=0.064)
	T2D	–0.035 (p=0.731)	–0.116 (p=0.262)	–0.156 (p=0.122)	–0.194 (p=0.053)	–0.194 (p=0.053)	0.047 (p=0.643)	**–0.210 (p=0.036)**	–0.138 (p=0.171)	–0.128 (p=0.204)
	All	0.009 (p=0.905)	–0.025 (p=0.742)	–0.101 (p=0.173)	–0.072 (p=0.336)	–0.044 (p=0.569)	0.029 (p=0.696)	**–0.175 (p=0.018)**	–0.146 (p=0.050)	–0.113 (p=0.128)
